# Content Validity, Feasibility, and Acceptability of the Neurosense PremmieEd Programme, a South African Premature Parenting Education Intervention for the NICU Parent: A Hybrid Focus Group Discussion Method

**DOI:** 10.3390/children12111502

**Published:** 2025-11-06

**Authors:** Welma Lubbe, Kirsten A. Donald

**Affiliations:** 1 Division of Developmental Paediatrics, Department of Paediatrics & Child Health, Red Cross War Memorial Children’s Hospital, Neuroscience Institute, University of Cape Town, Cape Town 7700, South Africa; kirsty.donald@uct.ac.za; 2 NuMIQ Research Focus Area, Faculty of Health Sciences, North-West University, Potchefstroom 2531, South Africa

**Keywords:** programme development, programme design, programmes, premature infant, parent educational interventions, neuroprotection, hybrid focus group

## Abstract

**Highlights:**

**What are the main findings?**

- The NeuroSense PremmieEd programme was adapted for South African NICU parents using a hybrid focus group method and found to be feasible, acceptable, and culturally relevant.
- Iterative feedback from mothers, clinicians, and experts led to improvements in content clarity, emotional sensitivity, readability, and accessibility.

**What are the implications of the main findings?**

- Hybrid focus group approaches can effectively inform the development of parenting education programmes in resource-constrained NICU settings.
- Structured, culturally aligned educational interventions have the potential to support parents of premature infants, improving preparedness and engagement in care.

**Abstract:**

Background: Parent education is a key component of family-centred care in neonatal intensive care units (NICUs). It supports positive parent-infant interactions, reduces parental stress and anxiety, and contributes to shorter hospital stays. Objectives: This paper reports on the adaptation of a South African parenting education intervention for parents of premature infants in the NICU: the NeuroSense PremmieEd programme. The study aimed to demonstrate the programme’s content validity, feasibility, and acceptability for preterm parent–infant dyads in public hospital NICUs, using a hybrid focus group discussion (FGD) method. The programme was based on an existing intervention and informed by literature on the components of parenting educational programmes and empirical data on parental expectations. Methods: A qualitative, iterative refinement process was undertaken using hybrid-format FGDs. A conceptual FGD was held during the design phase, followed by two consensus FGDs after pilot testing (reported separately). Stakeholders included end-users (mothers), clinicians, an instructional designer, a neurodevelopmental care expert, and programme facilitators. Results: The first FGD reviewed draft version 0.1 of the programme, confirming content relevance and clarity, while recommending adjustments, such as module integration, cultural and language alignment, and visual aids to support comprehension. Version 0.2 was then ready for pilot testing (reported elsewhere). The second and third FGDs led to refinements addressing emotional sensitivity in terminology, improved layout and readability, and the addition of home care guidance. Stakeholders highlighted the potential use of low-cost digital formats to enhance accessibility and standardisation. These revisions informed the final version 0.3. Conclusions: The hybrid FGD approach enabled input from diverse and geographically dispersed stakeholders. The NeuroSense PremmieEd programme was found to be feasible and acceptable by both mothers and healthcare professionals, supporting its suitability for broader implementation in resource-constrained settings.

## 1. Introduction

### 1.1. Background

Preterm birth affects approximately 1 in 10 births globally [1] and presents a range of challenges for both infants and their parents [2,3]. Due to early separation from the intrauterine environment, preterm infants are exposed to inappropriate sensory stimulation for their level of maturity, which may lead to physiological instability, increased pain sensitivity, disrupted sleep, and feeding difficulties [4,5]. As these infants continue to mature while coping with medical complications, parents often experience grief, anxiety, guilt, and feelings of incompetence [4,6,7]. To address these challenges and improve both short- and long-term outcomes, numerous interventions have been developed over time, such as Family-Integrated Care (FICare) [8,9], Creating Opportunities for Parent Empowerment (COPE) [10,11], PremieStart [12], Parental Sensitivity Intervention (PSI) [13], and the Parent Sensitivity Programme, a modified version of the Mother–Infant Transaction Programme (MITP) [14].

The NeuroSense PremmieEd parenting programme is one such programme (developed in the South African context) underpinned by the Synactive Theory of Development [15], a theoretical model of how premature infants regulate and organise their behaviour through interactions across five interrelated subsystems, namely the autonomic, motor, state, attention/interaction, and self-regulation subsystems. It further hypothesises critical and sensitive windows for brain development, especially periods during which the preterm infant may be particularly at risk, and emphasises the infant’s need for individualised, responsive interactions to support subsystem stability. Family-Centred Care (FCC) aligns with the neurodevelopmental principles of the Synactive Theory and serves to guide the implementation of the programme.

FCC is a complex care philosophy guided by principles, programmes, services, and practices that include parent support, relational communication [16], parental education [16,17], involvement in decision-making, caregiving participation, bereavement support, and transition-to-home preparation [17]. An FCC approach has been shown to enhance parental psychological well-being, promote bonding and attachment, reduce NICU length of stay, improve infant outcomes, and increase satisfaction for both families and staff [17,18,19]. FCC provides the relational and institutional scaffolding that enables parents to become sensitive observers and co-regulators of their preterm infant’s behavioural cues, thereby contributing significantly to optimal infant development during the NICU stay [18,19] and optimising neurodevelopmental outcomes.

Despite its complexity, FCC implementation has been supported through frameworks such as the Newborn Individualized Developmental Care and Assessment Program (NIDCAP), which shifts the focus from task-oriented care to relational processes that increase family involvement [20,21].

Within the larger, system-wide frameworks, several structured FCC-based programmes have demonstrated measurable benefits. The COPE programme reduced NICU and hospital stays by 3.8 and 3.9 days, respectively [22], while Alberta’s Family Integrated Care (FICare) education component reduced the length of stay by 2.55 days [23]. The PSI improved paternal attachment and self-efficacy within the first week post-intervention [13].

In South Africa, Lubbe [24,25] developed best-practice guidelines for neurodevelopmental supportive care of preterm infants, with Guideline 9 specifically advocating for family education. An underpinning literature review [26] emphasised the value of empowering parents to read behavioural signs and stress cues in their infants. In the South African context, one online programme was available, known as Little Steps^®^ [27]. This programme covers topics including the NICU environment, infant care, medical conditions, and transition to home. However, it was developed with a technology access focus and is available in an online, self-directed format, including materials that, although contextual for South Africa, reflect a private healthcare setting. Little Steps^®^ was not formally validated and documented. As a result, access to this (and potentially also other similar programmes in different countries) remains less accessible for those with restricted financial and digital resources.

Since 80% of the South African population depends on the public sector for medical services, it was important to develop a contextual programme which is specific to the population utilising these services, ensuring materials are presented at the most suitable literacy level, reflecting the public sector setting more clearly, and utilising platforms which are accessible to mothers in this context.

While many parenting interventions include education as one element of broader support strategies, our study focused on parent education as a standalone intervention aimed at improving parental and neonatal outcomes in the NICU. Education, as a central pillar of FCC, has consistently shown benefits, such as stronger parent-infant interactions, improved parental confidence, and a better understanding of preterm infant behaviour [22]. It has also been shown to empower parents towards becoming more confident carers [28]. Evidence from Benzies et al. [23] and Rajabzadeh et al. [29] reports educational interventions as reducing parental stress during NICU stays and after discharge. In addition, structured parenting programmes that emphasise active participation rather than passive integration into routine care have been linked to enhanced neurodevelopmental outcomes [30], all of which support parenting education as a feasible, cost-effective mechanism towards improved neurodevelopmental outcomes in the preterm infant-parent dyad.

Most existing programmes, such as FICare (Canada) [23], COPE (United States) [22], and PSI (Taiwan) [13], were developed in high-resource settings. In contrast, our objective was to develop a feasible and contextually relevant intervention tailored to the South African public healthcare sector, with special consideration of staffing constraints.

### 1.2. Aim

This paper reports on the design phase of a study focused on adapting and validating a parenting education programme for preterm infant–parent dyads in South Africa. The study aimed to draw on evidence from the literature and empirical data on parental expectations to adapt the Little Steps^®^ programme into the NeuroSense PremmieEd programme for implementation in public hospital NICUs in South Africa and to establish the content validity, feasibility, and acceptability of the new intervention.

### 1.3. Setting

The study was conducted in South Africa’s North West province, within an upper-middle-income country [31] context. This setting provides care to preterm infants in the NICU on various care levels, ranging from intensive care to kangaroo mother care units. Gestational ages of preterm infants admitted to these units range between 28 and 37 weeks, and infants are singletons or multiples. Mothers in this study who utilised these hospital services were mostly single, with an education level ranging between high school and some tertiary training. These hospitals serve a population that is a combination of rural and urban, although the hospitals are situated within urban areas. The population (mothers) in this setting is from a lower socio-economic background, and their infants ranged from very low birth-weight to late-preterm infants receiving intensive care to kangaroo mother care (KMC) within the KMC wards.

The design team comprised healthcare professionals from provincial hospitals and subject matter experts from across the country. The hospitals, experiencing resource constraints on various levels, but especially on a human resource level, do not necessarily have access to large and diverse multidisciplinary teams, but the purposefully selected design team (see participants) brought rich and varied perspectives.

### 1.4. Participants

Purposefully selected participants formed a multidisciplinary development panel to review the content, design, and overall development of the educational intervention [32]. The panel included key stakeholders [33]: an academic expert in neurodevelopmental supportive care (NDSC), a NICU clinician, on-site social workers, an instructional designer, and programme end-users—mothers of preterm infants (<37 weeks) currently admitted to the NICU. Due to a small number of mothers participating in this phase of the study, they could not be subdivided according to their infants’ birth weight or gestational age.

Academics provided theoretical grounding, clinicians contributed bedside expertise, and mothers and staff offered contextual insights to improve feasibility and acceptability.

To ensure continuity, the same NDSC academic and instructional designer participated in both focus groups (FGDs), 1 and 2. Focus group discussion 1 included a NICU mother and a senior neonatal nurse, both of whom offered rich contextual and practical perspectives. FGD 2 was intended to engage mothers who had recently completed the pilot, but due to caregiving demands, they withdrew shortly before the session. As a result, FGD 3 was held with only mothers currently in the NICU. A hospital-nominated social worker from Site B, and trained by the researcher to facilitate the programme, attended FGD 2 in person.

## 2. Materials and Methods

The NeuroSense PremmieEd programme structure has been presented elsewhere [34], with this paper focusing on the design phase of programme development, highlighting the use of a hybrid FGD technique that was conducted first as a conceptual FGD, followed by a consensus FGD after piloting the programme.

We utilised the pre-designed Structured Parent Education Assessment Tool (SPEAT) (refer to Supplement S1) to guide the FGD and obtain feedback in a structured manner from participants in the FGDs.

SPEAT for FGD 1 included all elements of the programme and three options for each element: *(1) definitely to be included (i.e., evidence-based, feasible to implement, and welcomed), (2) maybe to be included (i.e., some evidence base, feasible to implement but with challenges and disadvantages, welcomed by the majority), or (3) not to be included (i.e., limited evidence, challenging to implement, welcomed by some participants) and add additional comments as necessary* (see Supplement S1). SPEAT for FGD 2 used the scoring of *(1)—agree without any change, (0)—changes suggested, with a consensus statement on the finalised, revised item* for each item (refer to Supplement S1).

### 2.1. Recruitment

Ethical approvals were obtained from the Health Research Ethics Committee of the University of Cape Town (UCT-647/2021), followed by North West provincial and hospital management approval, with annual renewals. Participants were purposively selected based on their potential contributions to the study and were invited via email or in person by a research assistant. They could respond to requests for more information or confirm their availability. The research assistant conducted the informed consent process and forwarded the participant details to the first author, who facilitated the FGDs.

### 2.2. Focus Group Discussions (FGD)

To support broad participation across geographically dispersed stakeholders, a hybrid-format FGD was used. This format, which combined in-person and online engagement, facilitated real-time interaction and group dynamics while addressing cost and scheduling constraints. Hybrid FGDs are supported in the literature as a valid method for generating robust qualitative insights. Dwyer et al. [35] note that virtual participation can enhance the depth of discussion, especially on emotionally charged topics, and that data quality across virtual and in-person FGDs is comparable.

Two hybrid and one in-person FGDs (with moms only) were conducted as part of a cyclical, consensus-driven process to review and refine the parenting educational programme.

Prior to the first FGD, panel members received an electronic document (version 0.1) comprising (1) findings from a systematic review of international NICU-based parent educational programmes; (2) empirical data on the educational needs of South African NICU mothers; and (3) a draft parenting educational programme, informed by both data sets and utilising content from the Little Steps^®^ website [36]. A stable internet connection and hybrid setup were coordinated by the research assistant. Cameras were used during introductions and then switched off to conserve bandwidth. Participants introduced themselves, agreed on ground rules, and provided informed consent, including verbal permission to record.

For FGD 1, participants included a researcher, a NICU clinical nurse, an instructional designer, and a NICU mother meeting in person, while the academic expert and second author joined online. The first FGD began with a presentation of the foundational evidence, stated above, also shared with participants prior to the session.

FGD 1: The session began with a 30 min presentation covering key findings from prior research phases. Clarifying questions were encouraged throughout, although participants expressed confidence in the material. This was followed by a one-off 60 min structured discussion, during which panel members reflected on the findings and suggested changes using the SPEAT (see Supplement S1).

Data were collected using the SPEAT, which guided the evaluation and refinement of the programme. In FGD 1, panel members rated each item using a 3-point scale (1, 2, or 3), while a binary scale (0 or 1) was used in FGD 2 to indicate final agreement or disagreement (see Supplement S1). The SPEAT served as an a priori thematic framework to structure the discussions and organise the feedback.

FGD 2 (healthcare professionals): Following pilot testing of version 0.2, the pilot results were presented at FGD 2 and 3. The format mirrored that of FGD 1. The initial design proposed a single FGD (version 0.2) involving both healthcare professionals and mothers. However, consistent with the unpredictable demands of the NICU, several mothers who had consented to participate were recalled to the unit at short notice. To accommodate the constrained schedules of healthcare professionals, the research team proceeded with their session as planned and subsequently arranged a separate FGD at a time suitable for the mothers. For analytical purposes, FGDs 2 and 3 were therefore treated as a single group when drawing conclusions. After a 12 min presentation, participants reflected on the findings and evaluated each programme item using the SPEAT, indicating suggestions for final refinements (see Supplement S1). This 46 min session was recorded via Microsoft Teams and transcribed using its integrated function and then cross-verified with field notes to ensure accuracy.

In both FGD 1 and 2, online participants frequently revisited earlier discussion points via the chat function, contributing thoughtful and reflective input.

FGD 3 (mothers): For the mothers, the material for each item was shared, and then they had the opportunity to immediately evaluate each programme item using the SPEAT. Although this session only included in-person participation, the session was still recorded via Microsoft Teams and transcribed as performed in FGD 2.

### 2.3. Data Analysis

All FGD sessions, including participant discussions, chat transcripts, and audio recordings, were exported and reviewed. Transcripts were carefully corrected against the recordings to ensure fidelity to the original dialogue and support data triangulation. All Setswana sections were checked for correct translation to English. Although the background presentations were also recorded, only data directly related to the programme content discussions were analysed to inform revisions.

A thematic analysis approach was applied. Transcripts were read repeatedly for familiarisation, and themes were extracted based on the categories identified previously and presented in the SPEAT. Themes and categories were checked by an independent co-coder, and discrepancies were resolved through discussion until consensus was achieved.

In FGD 1, no programme items were removed; however, the panel suggested that psychological changes, support, and maternal physical recovery be combined. Most items received constructive suggestions leading to refinement of layout, use of pictures, and a more conversational style through speech bubbles. The preterm health and infant care sections were accepted without changes (see Supplement S1). These refinements were incorporated into programme version 0.2 (for pilot testing). In FGDs 2 and 3, consensus was reached on all items, and proposal refinements were actioned, resulting in version 0.3 of the programme, ready for implementation.

## 3. Results

To accommodate participants’ geographical dispersion and time constraints, two hybrid-format and one face-to-face FGD were conducted. Five participants contributed to FGD 1, three to FGD 2 and three to FGD 3 (mothers only).

### 3.1. FGD 1: Initial Programme Review

Developed for resource-constrained South African public healthcare settings, NeuroSense PremmieEd was designed to be freely accessible. The draft included eight key topics: (a) the NICU environment, (b) infection prevention, (c) preterm infant health, (d) parental psychological changes and support, (e) physical changes in mothers, (f) infant care, (g) preterm infant behaviour, and (h) discharge and transitioning home. The researcher presented version 0.1 to the panel, who offered feedback to inform pilot testing.

The panel confirmed that version 0.1 appropriately reflected the essential programme content. The researcher’s field notes captured feedback, which was used to update and refine the programme in collaboration with the instructional designer (version 0.2) for pilot testing.

#### FGD 1: Feedback on Version 0.1 (Healthcare Professionals and Mothers)

Design and comprehension considerations: The instructional designer suggested using dialogue-style speech bubbles to guide interpretation and foster engagement. Participant YH (FGD1) recommended: “If you have a speech bubble of a grandparent that says something, a speech bubble of a child that says something… And—and maybe in the form of question and answer.” This was especially relevant for parents who might rely solely on visual content to understand and initiate communication with staff.

A participating mother with limited English proficiency, assisted by an interpreter, emphasised the importance of home-language delivery. As a result, all materials were translated into Setswana for the pilot. Her engagement underscored the ability of visual materials to convey key messages independent of text, as she also responded that she cannot read, but ‘They can do it using photos’ (Participant M, FGD1). In addition, the academic highlighted the need for contextual images when stating, ‘I think it is absolutely crucial that there’s more diversity in the baby, uh, pictures’ (Participant S, FGD1), while the mother’s reply seemed less focused on the need for diversity, as she replied, ‘All of them are fine’ when asked if she would prefer to see only babies that represent her ethnicity (Participant M, FGD1).

A key recommendation was to consolidate the sections on psychological changes, support, and maternal physical recovery into a single module on Parental Self-Care, improving coherence while retaining the essential content. However, also to divide information to reflect ‘more support for the moms while she’s in the unit, and support that the staff members can provide the moms, or information that we can give to the moms while she’s in the unit, versus information to friends and family on how they can support the moms’ (Participant AVW, FGD1).

Delivery format and scheduling: To improve digestibility, the content was reorganised into five sections: Section 1: NICU, infection prevention, and preterm health, Section 2: parental self-care, Section 3: preterm infant behaviour, Section 4: infant care (A: handling and positive touch, B: feeding), and Section 5: discharge and home transition.

The mode of delivery was suggested to include various modalities. Participant AVW (FGD1) stated, ‘But if we are going to look at making this more universal for the South African population in general, we would have to possibly consider different modes of delivery and duplication across those modes of delivery. So, either a video and a booklet, so a mom in a specific setting will choose the videos, and another mom in another setting will choose the booklet, or a combination of the two versus, or and, real-life lecturing. I think by merely just going to look at visual representation, it’s not gonna be as informative as somebody sitting down with a mom and answering all her questions that she might have about yawning’.

A lecture-based delivery supported by printed materials was supported by all FGD participants. In the absence of prior evidence for optimal delivery timing, the programme was structured over one week, with daily sessions limited to 60 min and scheduled between feeding times to support mother-infant bonding. These revisions led to the development of version 0.2, which was piloted with a cohort of mothers (described in a separate publication).

### 3.2. FGD 2 and 3: Feedback on Version 0.2 (After Pilot Testing)

The second (healthcare professionals only) and third (mothers only) FGDs aimed to refine the programme following pilot implementation. A summary of the pilot findings, focusing on feasibility and acceptability, was presented to guide the discussion. We provide a brief summary of the pilot study here. Sixty participants were enrolled in a three-arm sequential design: 20 mothers received standard care (Arm 1), 20 received an educational booklet for self-study (Arm 2), and 20 received the booklet plus a facilitated group session with a trained healthcare educator (Arm 3). All participants completed a knowledge test and NICU stress scale before and after the intervention. Changes were observed in Arm 3, with knowledge scores improving slightly, and stress scores increased the least between groups; however, differences in knowledge and stress score changes between groups were not statistically significant. Following the pilot, panel members reviewed version 0.2, accepted all content items, and suggested enhancements for clarity, cultural relevance, and usability.

Presentation and language: Panel members confirmed that the illustrations were clear and accessible, even for non-English speakers. As one participant noted, ‘It was very easy, especially because it has pictures to look at’ (Participant SW, FGD2). At the same time, a key issue raised was inconsistency in the language use on visual materials, such as Afrikaans signage appearing in Setswana versions. Participant YH (FGD2) highlighted this, stating, ‘Language use in visuals lacks inclusivity. Although it says wash your hands… everything is in Afrikaans. It might be helpful to have these [ward posters] also within the hospital, in a language that’s more accessible to members that will use it’. Notably, mothers did not highlight this. While this could suggest they did not feel empowered to request it, they were also not directly asked about receiving the intervention in their own language. To address this, the panel recommended full language alignment across all visual and written content.

Delivery mode: Participants supported adding supplementary lectures and, eventually, digital formats (e.g., videos and touchscreen materials), stated by Participant YH (FGD2) as “Videos can support delivery of content and can be made accessible through QR codes or online links”, although these enhancements were postponed until after content validation.

Terminology: Feedback from programme presenters on the infant behaviour section revealed emotional concerns. One mother (in the pilot study) found the term “stress cues” distressing, believing it implied that she had caused harm. Participant AW (FGD2) noted that: “I’m just wondering about the term stress… A softer word, maybe … ‘cause they’re thinking they’re doing something damaging to their baby and completely wrong’”. The panel suggested using gentler terms, such as “unsettled”, to prevent misinterpretation and emotional distress.

Readability and navigation: Suggestions included increasing font sizes, decreasing white space, and breaking dense sections into multiple pages (e.g., skin-to-skin contact and parental support information). “The pictures are quite very small. … the font can be a bit big. The colours is OK” (Participant SW: FGD2). The instructional designer noted that Setswana’s grammatical complexity might require layout adaptation, and highlighting keywords was recommended to aid comprehension. Participant AW (FGD2) highlighted: “Design inconsistencies between English and Setswana versions, redesign layout to improve readability and finding information”. The restructured Parental Self-Care module, now including a question-and-answer format, was well received for encouraging personalised discussion. “That is more a question and discussion type of thing” (Participant SW: FGD2). Panel members proposed adding cross-references between sections (e.g., linking signs of unsettled behaviour to calming strategies).

Content and delivery enhancements: Additional content on home-based infection prevention was recommended under the skin-to-skin section. “We can just say, ‘refer back to earlier content’” (Participant SW: FGD2). Scripted slides were praised for balancing fidelity with adaptability. The potential use of digital content (e.g., videos, discussion-style recordings), particularly in areas without trained facilitators, was revisited and well supported by mothers.

Implementation challenges: Practical limitations were acknowledged: staffing constraints meant that only one session per week could be delivered, limiting parental engagement. Mothers stated that they did not have time to review the material due to competing commitments with infant care; as one mom stated, “I was not able to read that book because I was having struggles with the baby, I had to go up and down checking on them because they were crying and not getting full from milk.” (Participant M: FGD3).

The panel supported shorter, more frequent sessions, since “Running everything over an hour can be a lot” (Participant SW, FGD2); however, facilitator availability remained a challenge. The use of low-cost technological solutions was again endorsed to support content delivery and retention, as mentioned above, as stated by the same participant: “the idea of having a video where things can be cemented in… or just re-explain… maybe a link to where they can get more information.”

**Figure 1:**
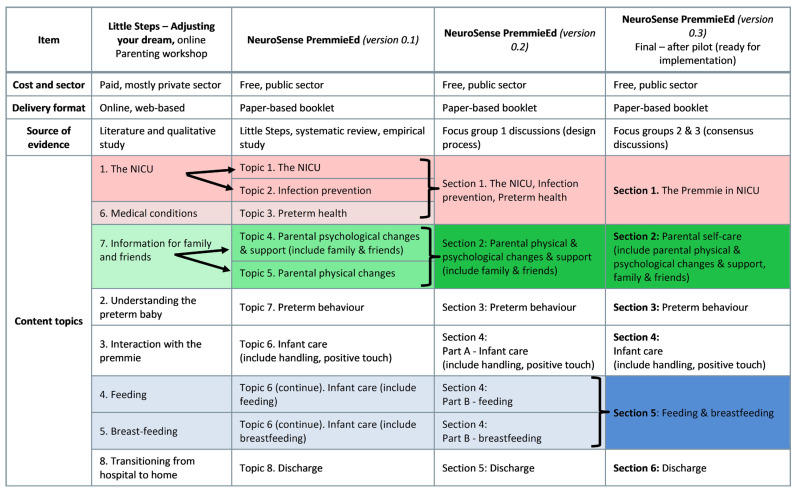
Programme changes across versions one to three.

Following this feedback, version 0.3 of the programme was finalised for implementation, with a recommendation to obtain funding towards the development of a digital format. A summary of the changes across the programme versions is presented in Figure 1.

## 4. Discussion

This study focused on establishing the content validity, feasibility, and acceptability of the NeuroSense PremmieEd programme, a South African parenting education intervention for caregivers of premature infants admitted to the NICU. To achieve this, we used a hybrid FGD format and engaged a multidisciplinary panel of stakeholders. The intervention progressed through three design iterations. Version 0.1 was informed by a systematic review, local empirical data, and culturally adapted materials from the Little Steps^®^ programme. FGD 1 confirmed the relevance of the proposed content and led to adaptations. These included the addition of more diverse illustrations and the use of more action-oriented text. Content on the NICU, infection control, and preterm health was consolidated into a single module, The Premmie in NICU. Similarly, content on parental physical and psychological changes was combined into a broader Self-Care module. These revisions resulted in version 0.2 of the programme. The programme content was found to align well with international programmes such as NIDCAP [37,38], COPE [22,39], and PSI [13] but is tailored to the South African context through linguistic and cultural adaptations, demonstrating content validity.

Following FGD 1, the programme was pilot tested (described elsewhere), and FGD 2 and 3 provided further refinement. The second discussion, which included both content reviewers and programme facilitators, was especially valuable, given the constraints to obtain feedback about feasibility in the South African public healthcare context. Unlike most other parenting programmes that depend primarily on NICU nurses [11,13,40,41,42,43,44,45], our findings showed that non-clinical staff, such as social workers and nurses not permanently stationed in the ward, could successfully deliver the programme. These facilitators found the materials clear and the slide scripts effective, even without prior intensive training in developmental care.

A key insight from all FGDs was the value of embedding psychological support within the educational framework. Mothers appreciated having a space to ask questions and described the sessions as both informative and therapeutic. This dual effect, offering emotional support alongside education, was particularly meaningful in a setting where access to formal psychological services is limited. This finding also explains the existence of many psychosocial support programmes [8].

Several practical issues were highlighted during FGD 2. Inconsistencies between visual materials and the language of delivery, such as ward posters in Afrikaans appearing in Setswana-language sections, highlighted the need for better visual-linguistic alignment. Facilitators also raised context-specific concerns, such as how to store breast milk without refrigeration. Visual aids showing feasible alternatives, such as cooler bags, were suggested. This type of information was not suggested in other literature but highlighted the importance of contextual considerations.

Facilitators observed that mothers who were initially reserved became more confident and engaged with their infants after the sessions, demonstrating the added value of in-person facilitation over written materials alone and suggesting that the educational intervention could also improve participants’ ability to respond to their infants’ behaviour and reduce stress.

Session timing and frequency emerged as areas requiring contextual flexibility. While the literature offers little guidance, preferences vary across facilities, with some favouring morning sessions and others preferring afternoon sessions. Both FGDs and supporting literature [46,47] suggest that shorter, more frequent sessions could improve engagement and retention. However, in resource-limited settings, clinical care understandably takes priority over parent education; therefore, one session of 60 min seems workable. Still, investing in accessible education may yield downstream benefits by empowering caregivers, improving neonatal outcomes, and easing clinical workloads over time. We also found that mothers presented as tired and listless, with a short attention span.

### Strengths and Limitations

This intervention was piloted in a predominantly Setswana-speaking community in South Africa’s North West Province. To ensure broader relevance, further piloting in other linguistic and cultural contexts is recommended. Although focused on the public sector, South Africa’s fluid healthcare landscape, where families often move between public and private care, warrants an evaluation of the programme’s applicability across sectors.

The alignment between international evidence and local needs suggests potential for wider implementation in other low- and middle-income settings, pending further validation. Exploring digital formats, such as videos, interactive platforms, or AI-assisted modules, could enhance accessibility, standardise content delivery, and reduce reliance on human resources by relieving pressure on bedside clinicians to provide structured education. Even in constrained settings, low-cost digital solutions may provide scalable and individualised support for parents.

While education remains a cost-effective and scalable intervention, a formal cost-effectiveness analysis is essential for scale-up planning. Future research should assess the programme’s broader impact on maternal mental health, infant outcomes, staff burden, and system efficiency. Long-term follow-up, particularly around neurodevelopmental outcomes, would offer valuable insights into the sustained impact of early parent education.

## 5. Conclusions

This study used a hybrid FGD approach to determine the content validity, feasibility, and acceptability of a parenting educational intervention for mothers of preterm infants in a South African NICU. Three FGDs were conducted, one during programme development and two following pilot implementation. This approach allowed for geographically dispersed participation, integrated transcription support, and additional input via chat features.

Participants included a diverse panel of key stakeholders: NICU mothers, an instructional designer, a neurodevelopmental care academic, NICU clinicians, and, in FGD 2, programme presenters. Their combined insights ensured that the content and delivery were both clinically relevant and contextually appropriate.

Findings indicate that the NeuroSense PremmieEd programme is feasible and acceptable to both mothers and healthcare professionals within the public sector of the North West Province, in South Africa. The educational programme holds the potential for broader use in similar resource-limited neonatal settings, where structured, accessible parent education can meaningfully support caregiver empowerment and improve care outcomes, and should be tested in similar settings.

## Data Availability

The data presented in this study are available on request from the corresponding author. The data are not publicly available due to copyright and ongoing development.

## Supplementary Materials

The following supporting information can be downloaded at: https://www.mdpi.com/article/10.3390/children12111502/s1, Supplement S1: Structured Parent Education Assessment Tool (SPEAT).
