# Supplementary material for: Content Validity, Feasibility, and Acceptability of the Neurosense PremmieEd Programme, a South African Premature Parenting Education Intervention for the NICU Parent: A Hybrid Focus Group Discussion Method
Source: Children (Basel). 2025 Nov 6;12(11):1502. doi: 10.3390/children12111502 (PMC12651853; doi:10.3390/children12111502)
Supplement: Supplementary file 1 [file children-12-01502-s001.zip › children-3883088-supplementary.pdf]

Supplement 1. Structured Parent Education Assessment Tool (SPEAT) used for FGD 1, 2, and 3.

Topics	Please reflect on the findings presented from the previous phases of this study. Based on these findings, what changes will you suggest for the parenting educational programme?	FGD 1				FGD 2 & 3		
		(1) definitely include	(2) maybe include	(3) not included	Comments	1 – agree without any change	0 – changes suggested, with a consensus statement on the finalised, revised item (comments)	Comments
**The NICU**	NICU environment, procedures, visitation, staff, equipment							
**Infection prevention**	Mostly handwashing							
**Preterm health**	Preterm infant symptoms/ condition / health/ appearance / danger signs (@home), temperature/ fever, medication, infant pain							
**Parental psychological changes & support**	Family dynamics (role of parents), fathers supporting role, emotional changes in mother (spiritual support), coping (grieve), positive parenting /interaction, attachment, adaptation to NICU (stress), self-care/ maternal wellbeing (birth experience), relaxation							
**Physical changes in mothers**								
**Infant care**	Positioning, diapers, bath & clothing, cord & eyes, sleep, infant massage, S2S (hold/ hug/ cuddle, feeding (BF, express)							
**Preterm behaviour**	Cues / interaction/ communication / calming, developmental care							
**Discharge**	Discharge preparation/ transition, follow-up, vaccination, resuscitation, playtime (stimulation)							
**Demonstrations**	Observing infant<br>Care of infant<br>Physical contact with infant							
**Key presenters**	MDT members<br>Nursing staff Psychologists							
**Characteristics**	Collaborative<br>Number of sessions: Not specified Frequency: When needed<br>Length: Lectures around 60min Time of day: Not specific							
**Method of delivery**	Visual (print) – lectures and F2F at bedside<br>Audio: MomConnect/ WA group Demonstrations at bedside							
